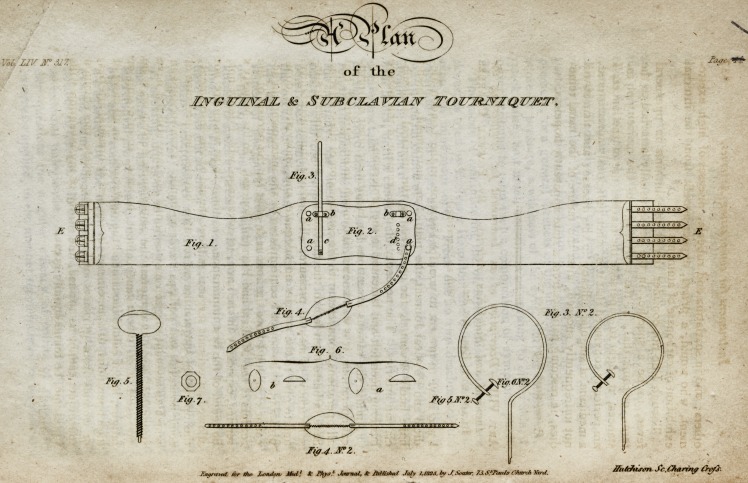# Description of an Instrument for Compressing the Inguinal and Subclavian Arteries

**Published:** 1825-07

**Authors:** Powell Charles Blackett


ART. VIII.—*Description of an Instrument for compressing the Inguinal and Subclavian Arteries.* By POWELL CHARLES BLACKETT, Esq. &c. &c.

[With an Engraving.]

I BEG leave to present to you, for insertion in the London Medical and Physical Journal, the plan of an instrument, which I have lately invented, for compressing the inguinal and subclavian arteries. The necessity of an instrument of this nature has long been felt and lamented in the profession.

Amidst the various inventions for facilitating other chirurgical operations, it is well known, and much to be regretted, that all operations, connected with the compression of these arteries, have hitherto been left to the unsteadiness, and sometimes insufficiency, of manual pressure. To remove this uncertainty, and to give to the arteries an adequate and undeviating compression, is the object I contemplated in the instrument, an account of which I have the honour to offer for the perusal of the readers of the Medical and Physical Journal. In his Majesty's service, especially in the navy, its advantages will, I doubt not, be found of the first importance. In those ships which are not allowed an assistant surgeon, without the aid of this instrument, the operating surgeon has no resource but in the pressure of some unskilful, and perhaps wavering hand. Even in ships in which an assistant surgeon may be found, he may still not have strength of nerve to compress the artery sufficiently; or, if he possessed it in the commencement, he may, from cramp or fatigue, lose it before the close of the operation. In all cases of high operations, of aneurisms of the femoral or brachial arteries, or wounds from gunshots or lacerations, where the common tourniquet cannot be applied, the utility of my invention will, I presume, be found equally great.

*27th April,* 1825.

*Description of the Plate.*

Fig. 1. A piece of broad silk webbing, hollowed out to fit exactly the nates, and underneath the arms, bound with silk ferret.

2. A plate of iron or steel.

3. Springs for stopping the inguinal and subclavian arteries.

4. A cap for the opposite shoulder, to which the spring is applied; with holes to fasten on the studs, and lined with chamois leather.

5. The tourniquet for increasing the pressure.

6. The form of the pads:—*a*, the femoral; *b*, the subclavian.

7. A turnscrew.

*a a a a*. Four studs for applying and fixing the shoulder-cap.

*b b*. Two sockets for receiving the springs.

*c*. The position of springs behind; to be downwards for the inguinal, and upwards for the subclavian.

*d*. Screw-holes for altering the length of springs.

*e*. Straps and buckles to fasten round the body.

Fig. 3, No. 2.<br>Fig. 5, No. 2.<br>Fig. 6, No. 2. } The form of the springs, tourniquets, and pads, when applied.

Length of the back-plate, $8\frac{1}{4}$ inches; width, $4\frac{1}{2}$ inches; thickness, $\frac{1}{8}$th inch.

Length of spring for inguinal, 22 inches; length of subclavian ditto, $19\frac{1}{2}$ inches.

Diameter of inguinal, from the end of the spring to the knee of ditto, $4\frac{1}{8}$ inches.

Diameter of subclavian, from ditto to ditto, $3\frac{1}{2}$ inches.

Length of the tourniquet, $3\frac{1}{2}$ inches.

Size of oval pad for the inguinal artery, $2\frac{1}{4}$ inches; width of ditto, $1\frac{1}{2}$ inch; height of a cone, $\frac{1}{4}$th inch.

Size of oval pad for the subclavian artery:—length, 2 inches; width, $1\frac{1}{8}$ inch; and to form the height of a cone, $\frac{3}{4}$th inch.

Length of large-sized inguinal spring, 26 inches.

Diameter of ditto, from the end of the spring to the knee of ditto, 6 inches.

## Figures and Tables

**Fig. 3. Fig. 1. Fig. 2. Fig. 5. Fig. 7. Fig. 4. Fig. 6. Fig. 4. No. 2. Fig. 5. No. 2. Fig. 6. No. 2. Fig. 3. No. 2. f1:**